# Obstetrical Teaching

**Published:** 1851-12

**Authors:** 


*Obstetrical Teaching.*—There is no single subject connected with clinical teaching, which, to us, seems more important than that of practical instruction in operative midwifery, and we are pleased to introduce to the notice of our readers, the fact that, in New York and Philadelphia, this subject has claimed the attention of competent private practitioners, who have entered upon the work with an interest and zeal which ought to be encouraged by a good attendance of pupils. Dr. Augustus K. Gardner, of New York, proposes, during the coming winter, to give a series of demonstrations on this branch of medical science, by which the student may learn from the manikin, and by actual experience, such obstetrical operations as he will meet with in his subsequent practice. Dr. Gardner has made such arrangements that he will be enabled to teach, by the appearances of the breast and nipple, the diagnosis of pregnancy; by the *toucher* and *ballottement*, the date of pregnancy; by the stethoscopic signs, the condition of the fœtus, &c. Manipulations with the hand upon the manikin and the living subject, are the only true means of gaining an available knowledge of obstetrical science; and these may be obtained of Dr. G. for five dollars — for a single course.

We would mention, also, the Obstetric Institute of Philadelphia, which has grown up under the fostering care of Dr. Joseph Warrington, of that city. We well remember the instructions we received under the auspices of this institution, and when troubled with a difficult case of labor, we recur, almost invariably, to the principles there received, and have never yet found them unavailing in the trying hour.

The lessons of the professor in the lecture-room are put in practice under the more private and social teachings of the Institute; and, while the former are necessary to prepare the soil and scatter the seed, the latter are the gentle rains which revive and invigorate the tender germ, bringing it forward from growth to growth, till a harvest of experience is laid by in the mind of the young practitioner, which relieves him of embarrassment when separated from the counsel of instructors, and thrown upon the world to find his way to honor and usefulness.

May these twin sisters, in the sister cities, vie with each other in promoting sound obstetrical learning, and eventually receive the full accomplishment of their anticipated reward.— *New Jersey Med. Reporter.* *(Editorial.)*

